# Learning from Biodegradable Coronary Stents: Future Directions for TPVR Biodegradable Stents

**DOI:** 10.3390/ijms27167172

**Published:** 2026-08-11

**Authors:** Zhaoyang Ye, Nina Sophie Pommert, David Meier, Stephanie L. Sellers, Jakob Christoph Voran, Oliver J. Müller, Derk Frank, Tim Attmann, Gregor Warnecke, Thomas Puehler, Georg Lutter

**Affiliations:** 1 Department of Cardiac Surgery, University Hospital Schleswig-Holstein (UKSH), 24105 Kiel, Germany; 2 German Centre for Cardiovascular Research (DZHK), Partner Site North, 20251 Hamburg, Germany; 3 Department of Cardiac and Thoracic Vascular Surgery, University Hospital Schleswig-Holstein (UKSH), Luebeck Campus, 23562 Lübeck, Germany; 4 Department of Cardiology, Lausanne University Hospital and University of Lausanne, 1015 Lausanne, Switzerland; 5 Centre for Cardiovascular Innovation, University of British Columbia, Vancouver, BC V5Z 1M9, Canada; 6 Cardiovascular Translational Laboratory, Providence Research & Centre for Heart Lung Innovation, Vancouver, BC V6Z 1Y6, Canada; 7 Centre for Heart Valve Innovation, St. Paul’s Hospital, Vancouver, BC V6Z 1Y6, Canada; 8 Department of Internal Medicine III, Cardiology, Angiology and Intensive Care Medicine, University Hospital Schleswig-Holstein (UKSH), 24105 Kiel, Germany

**Keywords:** bioresorbable stent, biodegradable stent, transcatheter pulmonary valve replacement, coronary bioresorbable stent, magnesium–zinc alloy, biodegradable metal, right ventricular outflow tract, tissue-engineered heart valve

## Abstract

Bioresorbable stents (BRS) have been explored in cardiovascular intervention to provide temporary mechanical support while reducing long-term foreign material. The coronary experience has shown both the potential and the limitations of this strategy. First-generation polymeric stents demonstrated feasibility but were limited by thick struts, insufficient radial strength, delayed healing, and increased scaffold thrombosis. In contrast, metallic bioresorbable platforms improved mechanical performance, but each material system still faces trade-offs between strength, degradation rate, and biological response. Transcatheter pulmonary valve replacement (TPVR) may represent a clinically meaningful setting for renewed BRS development. Patients with congenital heart disease often require repeated pulmonary valve interventions over a lifetime, and permanent metallic frames may increase cumulative implant burden and complicate future treatment. However, TPVR imposes distinct requirements, including large-diameter expansion, stable anchoring, fatigue resistance, controlled degradation, and leaflet-frame integration. This review summarizes the lessons learned from coronary BRS, discusses material considerations for TPVR-oriented stent design, and evaluates current preclinical evidence for bioresorbable and regenerative pulmonary valve platforms. Particular attention is given to magnesium–zinc alloys as a tunable material strategy for future bioresorbable TPVR frames. Although direct evidence for fully bioresorbable metallic TPVR devices remains limited, this approach provides a rational framework for next-generation pulmonary valve intervention.

## 1. Introduction

Permanent metallic stents have been central to modern cardiovascular intervention. In coronary artery disease, metallic drug-eluting stents have reduced restenosis and improved procedural safety. In structural heart disease, metallic valve frames have enabled transcatheter valve replacement in patients who previously required repeated open-heart surgery. Despite these achievements, permanent metallic implants are not without limitations. Long-term metallic caging may impair local tissue compliance, contribute to chronic foreign-body responses, complicate future reintervention, and create cumulative implant burden in patients requiring multiple procedures over a lifetime [1,2,3].

Bioresorbable stents (BRS) were developed to address these concerns. The basic concept is straightforward: the stent provides temporary mechanical support during early healing and then gradually degrades after its structural function is no longer required. The coronary field has provided the most extensive clinical experience with this approach [4]. Early polymeric BRS, especially poly-L-lactic acid-based platforms, showed that complete stent resorption was feasible.

However, clinical trials also exposed important weaknesses, including thick struts, limited radial strength, delayed endothelialization, impaired deliverability, and increased scaffold thrombosis compared with contemporary metallic drug-eluting stents [5,6]. These findings did not invalidate the bioresorbable concept, but they showed that material selection, stent geometry, degradation kinetics, and implantation technique are decisive for clinical success [1].

Recent translational reviews have emphasized that next-generation biodegradable stents require simultaneous optimization of material composition, stent architecture, drug delivery, degradation control, and clinical implantation strategy rather than improvement of a single parameter alone [7].

The limitations of polymeric BRS have shifted attention toward bioresorbable metallic stents. Magnesium-, iron-, and zinc-based systems have all been investigated. Magnesium alloys offer better mechanical performance than polymers and have favorable biocompatibility, but rapid corrosion may cause premature loss of radial support [8]. Iron-based stents provide high mechanical strength but degrade slowly, leading to prolonged retention of corrosion products [9]. Zinc has a more moderate physiological corrosion profile, although pure zinc lacks sufficient strength for many load-bearing cardiovascular applications [10]. These trade-offs have encouraged alloy-based strategies. In particular, magnesium–zinc systems may provide a tunable balance between mechanical strength, ductility, degradation behavior, and biological response [11,12].

Although coronary intervention remains the main source of clinical evidence for BRS, the concept may also be relevant to valvular heart disease. Transcatheter valve devices rely on metallic frames to provide anchoring, radial support, and leaflet fixation. This requirement is especially important in transcatheter pulmonary valve replacement (TPVR), which is widely used to treat right ventricular outflow tract (RVOT) dysfunction in patients with repaired congenital heart disease, failing right ventricle-to-pulmonary artery conduits, and degenerated bioprosthetic valves [13]. Many of these patients are children, adolescents, or young adults, and repeated interventions are often expected over their lifetime. In this setting, permanent metallic frames may lead to cumulative metal burden after valve-in-valve, restrict future options, and limit adaptability to somatic growth in pediatric patients [3].

This review summarizes the clinical lessons learned from coronary bioresorbable stents, compares major degradable material platforms, and discusses their potential application in valvular interventions. Particular emphasis is placed on TPVR as an emerging valvular application and on magnesium–zinc alloys as promising candidate materials for next-generation bioresorbable pulmonary valve frames.

## 2. Clinical Applications and Evolution of Coronary Bioresorbable Stents

The coronary BRS field has moved through two major phases: an early polymer-dominant era and a later metallic bioresorbable stent era. This chronological transition highlights a central principle: bioresorbability alone is insufficient unless mechanical support, strut design, degradation kinetics, and tissue healing are properly balanced.

### 2.1. The Polymeric Era

The first clinically important coronary BRS platforms were polymer-based. The Igaki–Tamai scaffold, composed of poly-L-lactic acid (PLLA), provided early proof that a coronary stent could be implanted and gradually resorbed in humans [14]. This concept was later developed into drug-eluting polymeric stents, most notably the Absorb bioresorbable vascular scaffold. Absorb was designed to provide temporary radial support, release everolimus, and then disappear over time.

However, the intrinsic mechanical weakness of PLLA (poly-L-lactic acid) required relatively thick struts, approximately 150–157 μm, to provide adequate radial strength. This bulky strut profile increased flow disturbance, delayed endothelialization, and made implantation technique particularly important [15]. In the ABSORB III trial, three-year target lesion failure was higher with Absorb than with metallic everolimus-eluting stents (13.4% vs. 10.4%), and definite or probable device thrombosis was also higher (2.3% vs. 0.7%) [16]. Similarly, the ABSORB II randomized trial failed to demonstrate late vasomotor superiority and reported less favorable device-oriented outcomes with Absorb at three years, reinforcing concerns that first-generation PLLA stents could not match contemporary metallic everolimus-eluting stents in routine lesion settings [17]. A patient-level pooled analysis of the ABSORB randomized trials confirmed this safety signal, reporting higher three-year target lesion failure (11.7% vs. 8.1%) and scaffold thrombosis (2.4% vs. 0.6%) with Absorb compared with metallic everolimus-eluting stents [6]. These data led to a marked decline in clinical use, and Absorb was commercially discontinued in 2017. The AIDA trial further extended these observations to routine PCI practice, showing a higher rate of device thrombosis with Absorb than with metallic everolimus-eluting stents, which strongly influenced the subsequent decline in clinical use [18].

Other polymeric platforms attempted to overcome some of these limitations. The FANTOM scaffold used iodinated tyrosine polycarbonate to improve radiopacity and reduce strut thickness to approximately 125 μm. In the FANTOM II trial, one-year outcomes showed low adverse event rates, with target lesion failure-like device-oriented events around 4.2% and definite/probable scaffold thrombosis around 0.4% [19]. Later five-year follow-up suggested acceptable long-term safety in selected patients, although the evidence remained non-randomized and limited in scale [20]. Overall, the polymeric era demonstrated that complete resorption was feasible but also showed that weak radial strength, thick struts, and delayed healing could offset the theoretical benefits of a “leave nothing behind” strategy. A large meta-analysis of randomized trials confirmed that the adverse-event signal was most pronounced during the stent resorption period, supporting the view that early stent design, strut thickness, and implantation technique were central determinants of clinical outcome [21].

### 2.2. The Metallic Era

The limitations of polymeric BRS prompted a shift toward bioresorbable metallic stents. Compared with polymers, biodegradable metals provide higher mechanical strength, better ductility, and the potential for thinner struts. Magnesium-based stents were the first metallic BRS platforms to reach substantial clinical evaluation.

The earliest clinical magnesium stent, tested in the PROGRESS-AMS trial, demonstrated the feasibility of absorbable metal scaffolding in human coronary arteries but also revealed important limitations, including early recoil and restenosis related to rapid loss of mechanical support [22]. Later magnesium platforms improved stent design, drug elution, and degradation control. Magmaris, derived from the DREAMS 2G magnesium alloy platform, is a sirolimus-eluting magnesium stent with a strut thickness of approximately 150 μm. Preclinical degradation mapping of magnesium stents showed that second-generation designs achieved more controlled structural resorption than earlier magnesium platforms, supporting the importance of alloy processing and surface design in delaying premature mechanical loss [23]. In the BIOSOLVE-II five-year follow-up, target lesion failure was 8.0%, with no definite or probable scaffold thrombosis reported [24]. In the larger BIOSOLVE-IV registry, the first 400 patients showed a 12-month target lesion failure rate of 4.3% and a low definite/probable scaffold thrombosis rate of approximately 0.5% [25].

Iron-based stents represent another metallic strategy. The nitrided iron-based IBS coronary scaffold has an ultrathin strut thickness of approximately 53 μm, enabled by the high mechanical strength of iron. In the IBS-FIM trial, three-year target lesion failure was 6.7%, and no definite or probable scaffold thrombosis was reported [26]. However, the major limitation of iron-based stents is slow degradation, with prolonged persistence of iron corrosion products. Thus, iron-based BRS illustrate the opposite problem to magnesium: excellent mechanical strength but insufficient resorption speed.

### 2.3. Emerging Zinc and Magnesium–Zinc Alloy Systems

Zinc-based materials have gained attention because their physiological corrosion rate is generally slower than magnesium and faster than iron. This intermediate degradation profile is attractive for cardiovascular stents. However, pure zinc has insufficient mechanical strength for load-bearing stent applications, leading to the development of Zn-based alloys such as Zn-Cu, Zn-Mn, and Zn-Ag.

Large-animal vascular studies support the potential of Zn-based systems. In a two-year porcine coronary implantation study, biodegradable Zn-Cu stents maintained early structural integrity and showed progressive degradation, with approximately 28% residual stent volume at 24 months and no severe inflammatory reaction [27]. These findings suggest that zinc alloys may provide more gradual degradation than magnesium while avoiding the long persistence of iron-based stents.

Magnesium–zinc alloy systems are particularly relevant because zinc can refine the magnesium microstructure, improve strength and ductility, and modulate corrosion behavior. Preclinical magnesium alloy systems containing zinc, such as Mg-Nd-Zn-Zr, have shown favorable vascular responses when combined with polymeric drug-eluting coatings. In a porcine coronary model, a PDLLA/rapamycin-coated Mg-Nd-Zn-Zr stent demonstrated acceptable biosafety, inhibition of neointimal hyperplasia, and evidence of re-endothelialization at six months [28]. Although these findings are still preclinical, they support the rationale for further development of Mg-Zn-based bioresorbable metallic platforms.

**Figure 1:**
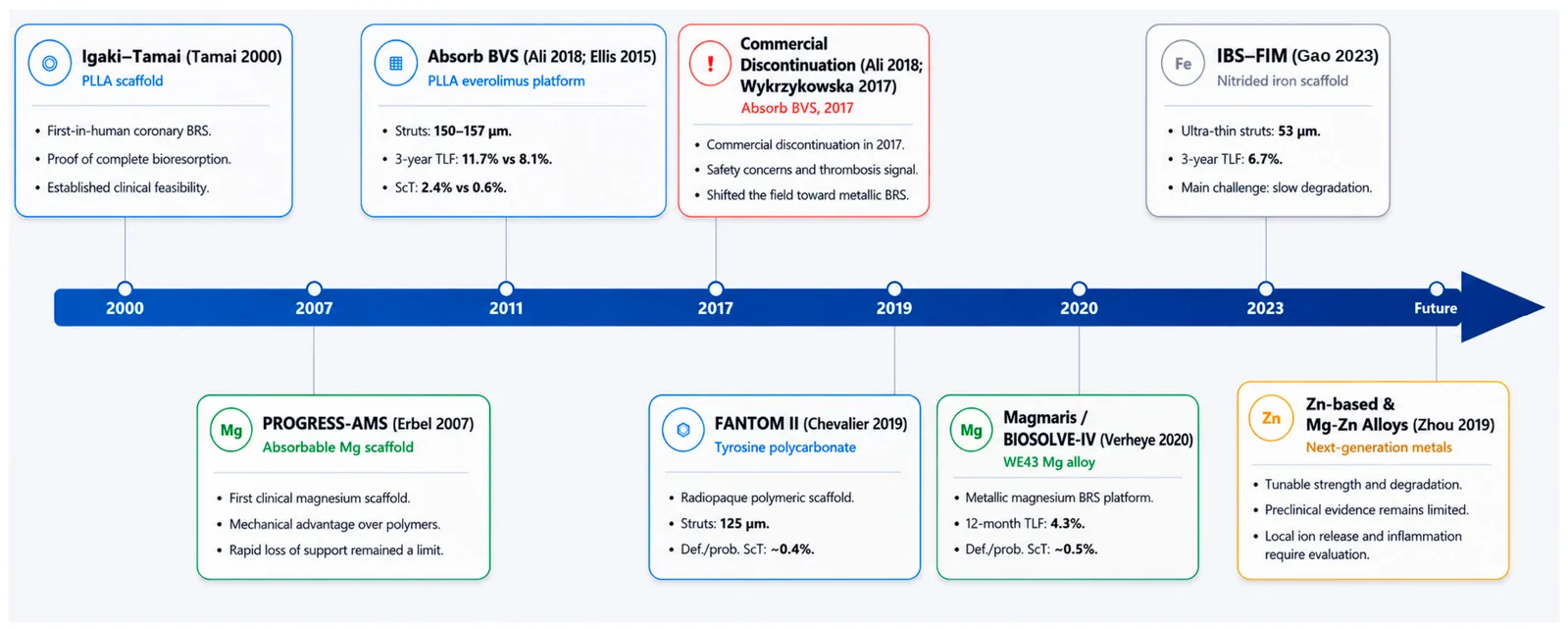
Chronological evolution of coronary bioresorbable stents [6,14,15,18,19,22,25,26,27]. Abbreviations: BRS, bioresorbable stents; BVS, bioresorbable vascular scaffold; PLLA, poly-L-lactic acid; ScT, scaffold thrombosis; TLF, target lesion failure.

The coronary BRS experience underscores several design principles that are highly relevant to future cardiovascular applications. Early mechanical integrity remains a prerequisite for safe and effective implantation, whereas strut thickness and stent geometry critically shape the subsequent healing response and thrombotic profile. Equally important, stent degradation should proceed in concert with tissue repair, ensuring that structural support is not withdrawn before biological stabilization is achieved. Accordingly, material choice must be adapted to the target anatomy and local loading conditions. These principles become even more stringent when moving from small coronary arteries to large-diameter valvular structures, in which radial force, crimpability, fatigue durability, and predictable degradation must be optimized under far more demanding biomechanical constraints. The chronological evolution of these platforms is summarized in Figure 1, and representative clinical and preclinical studies are presented in Table 1 and Table 2, respectively.

The timeline summarizes the transition from early polymeric coronary bioresorbable stents to bioresorbable metallic platforms, including magnesium- and iron-based systems. Key milestones include the Igaki–Tamai PLLA stent, PROGRESS-AMS magnesium scaffold, Absorb BVS commercialization and discontinuation, Magmaris registry data, and recent iron-based stent trials.

## 3. Clinical Rationale for Bioresorbable Stents in TPVR

Transcatheter pulmonary valve replacement (TPVR) is one of the most clinically relevant valvular settings for the translation of bioresorbable stent concepts. Unlike coronary intervention, where bioresorbable stents must function in small-caliber vessels with a low tolerance for bulky struts, TPVR is performed in larger right ventricular outflow tract (RVOT) structures and mainly requires stable valve anchoring, sealing, and leaflet support. This difference does not make the engineering easier, but it changes the clinical rationale for using a temporary stent.

TPVR was first introduced as a less invasive treatment for dysfunctional right ventricle-to-pulmonary artery conduits [13]. It is now used in selected patients with repaired congenital heart disease, failing RVOT conduits, and degenerated surgical bioprostheses. These patients are often children, adolescents, or young adults. Many of them will require more than one pulmonary valve intervention during their lifetime [30,31]. Therefore, the long-term consequences of each implanted frame are clinically important.

Current TPVR devices have achieved meaningful clinical success. Balloon-expandable systems, such as the Melody valve and SAPIEN valves, provide reliable treatment for conduit failure and bioprosthetic valve dysfunction [3,32]. Self-expanding platforms, including Harmony, Venus *p*-Valve, and Pulsta, have expanded treatment options for larger or more irregular native RVOT anatomies [33,34,35]. Adaptive pre-stenting systems, such as Alterra, further extend the use of transcatheter therapy by creating a more suitable landing zone for valve implantation [36]. These platforms have established TPVR as an important alternative to repeat surgical pulmonary valve replacement.

A bioresorbable TPVR frame would aim to provide temporary radial support during the early phase after implantation and then gradually degrade after valve stabilization and tissue integration. In principle, such a design could reduce long-term foreign material, facilitate future reintervention, and better align TPVR with the broader concept of in situ tissue-engineered heart valves [37,38]. This concept is particularly attractive in congenital heart disease, where the goal is not only short-term hemodynamic correction but also long-term management across childhood, adolescence, and adulthood.

**Figure 2:**
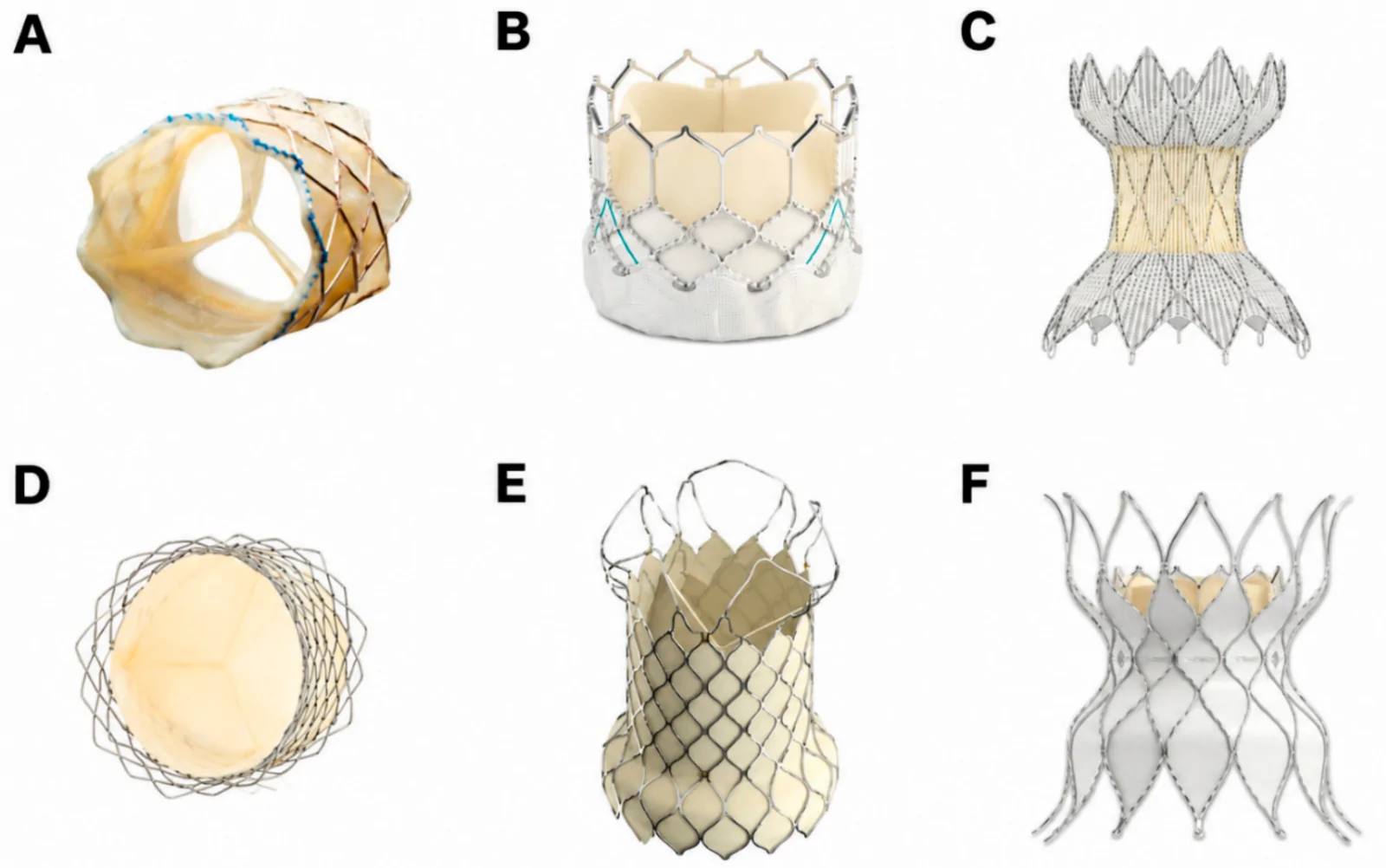
Representative commercially available transcatheter pulmonary valve replacement (TPVR) systems. (**A**) Melody transcatheter pulmonary valve (Medtronic), a balloon-expandable bovine jugular vein valve mounted on a platinum–iridium stent frame, representing the first clinically approved TPVR device, with the permission of reproduction by Medtronic Inc, Minneapolis, MN, USA. (**B**) SAPIEN 3 transcatheter heart valve (Edwards Lifesciences), a balloon-expandable bovine pericardial valve mounted on a cobalt–chromium frame, widely adopted for dysfunctional right ventricle-to-pulmonary artery conduits and failed bioprosthetic valves, with the permission of reproduction by Edwards Lifesciences Inc, Irvine, CA, USA. (**C**) Harmony transcatheter pulmonary valve (Medtronic), a self-expanding nitinol-based system specifically designed for large native right ventricular outflow tracts (RVOTs), with the permission of reproduction by Medtronic Inc, Minneapolis, MN, USA. (**D**) Pulsta transcatheter pulmonary valve (TaeWoong Medical), a self-expanding symmetric nitinol valve system developed for treatment of pulmonary regurgitation in enlarged native RVOT anatomy, with the permission of reproduction by TaeWoong Medical Inc, Gimpo-si, Republic of Korea. (**E**) VenusP-Valve (Venus MedTech), a self-expanding porcine pericardial valve mounted on a nitinol frame with flared proximal and distal ends for anchoring within dilated RVOTs, with the permission of reproduction by Venus MedTech Inc, Hangzhou, China. (**F**) Alterra Adaptive Prestent system (Edwards Lifesciences), a self-expanding nitinol docking platform designed to create a stable landing zone for implantation of a 29 mm SAPIEN 3 valve in large native RVOT anatomies, with the permission of reproduction by Edwards Lifesciences Inc, Irvine, CA, USA.

However, TPVR should not be regarded as a simple enlargement of coronary BRS technology. The pulmonary valve frame must tolerate large-diameter expansion, maintain anchoring in variable RVOT anatomy, interact safely with biological or polymeric leaflets, and resist cyclic deformation during cardiac motion. These demands are different from those in coronary arteries and require specific design criteria. Thus, TPVR represents a promising but technically demanding field for bioresorbable stent development. Representative commercially available TPVR systems are shown in Figure 2.

## 4. Design Requirements for Translating Bioresorbable Stents to TPVR

Native or surgically repaired RVOTs may be tubular, dilated, patched, calcified, or markedly asymmetric. Therefore, a bioresorbable TPVR frame must not only expand to a large diameter but also create stable anchoring and sealing in a non-uniform anatomical environment [3].

This size difference has direct consequences for material selection and device design. A TPVR stent must be crimped into a catheter-based delivery system and then expanded to a large final diameter without fracture, excessive recoil, or loss of geometric symmetry. These requirements place greater emphasis on ductility and controlled plastic deformation than is usually needed for coronary stents. The clinical experience with first-generation polymeric coronary BRS showed that limited radial strength and thick struts can compromise performance even in small vessels [1,5,6].

Anchoring is another major distinction between coronary and pulmonary valve applications. A coronary stent mainly supports the vessel wall. A TPVR frame, however, supports a valve. It must maintain the circularity of the valve orifice, prevent migration, limit paravalvular leakage, and preserve leaflet coaptation. Any loss of radial force, local collapse, or asymmetric degradation may affect valve competence. The frame and leaflets are mechanically coupled.

The RVOT also exposes the frame to repeated dynamic loading. Unlike a coronary stent, which mainly faces radial compression and pulsatile vessel motion, a pulmonary valve frame experiences cyclic deformation from cardiac contraction, respiratory motion, and repetitive leaflet opening and closure. Over a single year, the device is exposed to tens of millions of cardiac cycles. Permanent TPVR frames have already shown that stent fracture and structural fatigue can be clinically relevant, especially in anatomies exposed to compression or high mechanical stress [39]. A bioresorbable frame adds another layer of complexity because its mechanical properties change during degradation. Fatigue resistance must therefore be evaluated not only at implantation but also during progressive corrosion or polymer hydrolysis.

The degradation profile should match the biological time course of valve stabilization and tissue integration. If degradation is too rapid, radial support may be lost before the frame is incorporated into surrounding tissue, increasing the risk of recoil, migration, or valve dysfunction.

These requirements explain why the material criteria for TPVR differ from those for coronary BRS. Thin struts and low thrombogenicity remain important, but they are not sufficient. A TPVR-oriented bioresorbable stent must combine crimpability, large-diameter expansion, radial strength, fatigue resistance, controlled degradation, and compatibility with valve leaflets. In this context, magnesium-, zinc-, iron-, and polymer-based systems should be evaluated not only by their general biodegradability but by their ability to meet the specific mechanical and biological demands of the pulmonary valve position [40]. The principal differences between coronary BRS and TPVR BRS are summarized in Table 3.

## 5. Material Considerations for TPVR-Oriented Bioresorbable Stents

Material selection for a bioresorbable TPVR frame should be guided by the mechanical and biological requirements of the pulmonary valve position. TPVR frames must provide large-diameter expansion, stable anchoring, leaflet support, fatigue resistance, and controlled degradation during tissue integration. Therefore, the suitability of a material cannot be judged only by its biodegradability. It must also maintain mechanical function during the early post-implantation phase. In vivo screening approaches for candidate bioabsorbable stent metals further indicate that degradation behavior, local tissue response, and retention of corrosion products should be evaluated together, because in vitro corrosion alone may not predict biological performance [41].

PLLA-based platforms demonstrated that complete stent resorption was feasible, but their low intrinsic mechanical strength required thick struts, which contributed to impaired deliverability, delayed healing, and higher scaffold thrombosis in coronary applications [1,5,6]. Modified polymers, such as iodinated tyrosine polycarbonate, improved radiopacity and allowed thinner designs, but the load-bearing capacity of polymeric materials remains limited compared with metals [19,20]. For TPVR, polymers may still be useful as leaflet materials, tissue-engineering matrices, drug carriers, or surface coatings. However, as the sole load-bearing frame, purely polymeric materials are unlikely to provide sufficient radial strength, ductility, and fatigue resistance for large-diameter transcatheter pulmonary valve devices.

Biodegradable metals offer a more relevant starting point for TPVR-oriented stent design [42]. Iron-based systems provide high mechanical strength and can support very thin strut architectures, as shown by the nitrided iron IBS stent in coronary intervention [26]. This strength is attractive for a pulmonary valve frame, where anchoring and fatigue resistance are critical. The main drawback is degradation. Iron and many iron-based alloys corrode slowly, and insoluble corrosion products may persist in tissue for a prolonged period [9,12,43]. In TPVR, such slow resorption may reduce the advantage of a bioresorbable design because the frame could behave functionally as a semi-permanent implant. Accelerating iron degradation through alloying or surface engineering is possible, but the long-term biological response to residual corrosion products remains a major concern.

Magnesium alloys are more clinically advanced among biodegradable metallic stents. Magnesium has favorable biocompatibility, relatively low density, and better mechanical performance than polymers [8,12]. The clinical experience with magnesium coronary stents suggests that magnesium-based frames can be implanted safely in selected vascular settings [22,24,25]. However, magnesium corrosion can be rapid in chloride-rich physiological environments, and premature loss of radial support remains a key limitation [8,44]. For TPVR, this issue is particularly important because the frame must remain stable while leaflet integration and tissue remodeling occur. Uncontrolled magnesium degradation may also produce local alkalinization and hydrogen evolution, which must be managed through alloy design, surface coating, and device geometry.

Zinc occupies an intermediate position between magnesium and iron. Recent reviews of biodegradable zinc alloys also emphasize that alloying and thermomechanical processing are necessary to overcome the low strength and limited ductility of pure zinc while preserving its favorable corrosion profile [45]. It degrades more slowly than magnesium and more rapidly than iron, which makes it attractive for bioresorbable cardiovascular stents [10,12,27]. Pure zinc, however, does not provide sufficient mechanical strength for many load-bearing applications. Zinc alloys, including Zn-Cu and related systems, have shown improved mechanical properties and encouraging vascular compatibility in preclinical models. In a porcine coronary model, Zn-Cu stents showed gradual degradation over two years and no severe inflammatory reaction [27]. However, excessive local Zn^2+^ release may cause concentration-dependent cytotoxicity, and the biological response varies with alloy composition and degradation behavior; therefore, potential local toxicity requires careful evaluation [45]. These findings support the biological potential of zinc-based stents. Nevertheless, zinc-based systems remain largely preclinical, and their fatigue performance and large-diameter expandability require further evaluation.

Magnesium–zinc-containing alloys are especially relevant for TPVR because they may combine the advantages of magnesium and zinc while reducing some limitations of each element. Zinc can strengthen the magnesium matrix, refine microstructure, and help tune corrosion behavior [11,12,28]. In preclinical vascular studies, Mg-Nd-Zn-Zr and Mg-Zn-Y-Nd systems have shown acceptable biosafety, reduced neointimal response, and favorable endothelialization when combined with suitable surface treatment or drug-eluting coatings [11]. These findings support the rationale for Mg-Zn-based systems as candidate materials for next-generation bioresorbable frames. A TPVR-oriented comparison of the candidate material classes is provided in Table 4.

## 6. Molecular and Cellular Responses to Bioresorbable Stent Degradation

The biological performance of a bioresorbable stent is shaped not only by its bulk degradation rate but also by the host foreign-body response, including acute inflammation, macrophage recruitment, foreign-body giant cell formation, and fibrotic encapsulation [46]. Therefore, surface chemistry, degradation products, topography, and local mechanical cues should be considered as immunomodulatory design variables rather than passive material properties [47]. Macrophage phenotype is particularly relevant for regenerative pulmonary valve concepts because a balanced transition from pro-inflammatory to pro-remodeling macrophage responses may support extracellular matrix deposition and constructive tissue integration [48]. For biodegradable metallic stents, corrosion products should be regarded as active biological cues rather than inert by-products. Released Mg^2+^, Zn^2+^, or Fe ions, together with local pH changes and corrosion-particle accumulation, may influence endothelialization, smooth muscle cell proliferation, inflammatory signaling, and extracellular matrix remodeling [49]. These effects are especially important in TPVR, where the stent must remain compatible not only with the RVOT wall but also with biological, polymeric, or tissue-engineered leaflets. Persistent inflammation or poorly controlled degradation may impair leaflet-frame integration and may increase the risk of fibrosis, calcification, or late valve dysfunction [50]. Therefore, future TPVR-oriented bioresorbable stents should be evaluated using combined mechanical, corrosion, endothelialization, macrophage-response, and calcification assays.

## 7. Preclinical Evidence for Bioresorbable and Regenerative Pulmonary Valve Platforms

Direct evidence for fully bioresorbable metallic stents in TPVR remains limited. To date, no Mg-Zn or other biodegradable metallic TPVR frame has been validated as an established clinical platform. Most available evidence comes from related but distinct fields: tissue-engineered pulmonary valves, decellularized pulmonary homografts, bioresorbable polymeric valve scaffolds, and transcatheter tissue-engineered heart valve models. Recent reviews of next-generation tissue-engineered heart valves have emphasized repair, remodeling, and regeneration capacity as key requirements for living valve substitutes, which aligns conceptually with the long-term goals of bioresorbable TPVR frames [51].

Early work showed that biodegradable scaffolds could support pulmonary valve tissue formation [52]. Shinoka et al. reported valve leaflet replacement using a biodegradable scaffold in a lamb model, providing one of the earliest in vivo demonstrations of scaffold-guided pulmonary valve tissue engineering [53,54]. Clinical experience with decellularized pulmonary homografts later showed that cell-free biological scaffolds can be used for pulmonary valve replacement in children and young adults, with encouraging early and mid-term outcomes [55,56,57]. These studies support the biological feasibility of remodeling-oriented pulmonary valve substitutes, although they are not transcatheter metallic BRS platforms.

More directly relevant to TPVR are transcatheter tissue-engineered valve studies. Driessen-Mol et al. reported catheter-based implantation of homologous off-the-shelf tissue-engineered heart valves in sheep, demonstrating in vivo remodeling and self-repair capacity in the pulmonary position [56]. Kluin et al. further showed that a bioresorbable elastomeric valve implant could undergo host cell infiltration, extracellular matrix deposition, and remodeling during 12 months of follow-up in sheep [58]. Emmert et al. demonstrated that computational modeling can guide tissue-engineered valve design and improve long-term in vivo performance in a translational sheep model [59].

These studies provide important biological and engineering evidence for regenerative pulmonary valve platforms. However, they do not yet establish the safety or durability of a fully bioresorbable metallic TPVR frame. For Mg-Zn-based TPVR development, the key unanswered questions remain mechanical rather than conceptual: whether the frame can be crimped, expanded to large RVOT diameters, maintain radial force during degradation, resist fatigue fracture, and integrate reliably with biological or polymeric leaflets. Representative evidence for bioresorbable and regenerative pulmonary valve platforms is summarized in Table 5.

## 8. Future Perspectives

The next phase of bioresorbable TPVR development should focus not simply on whether a stent frame can disappear, but on when its mechanical support can be safely withdrawn. The coronary experience shows that complete resorption alone does not guarantee late clinical benefit: the final 7-year COMPARE-ABSORB results did not demonstrate superiority over contemporary Everolimus-eluting stents after the expected resorption period [60]. Conversely, recent third-generation magnesium and ultrathin iron platforms have shown encouraging mid-term performance, although the magnesium evidence remains single-arm and the iron platform has only recently entered randomized clinical comparison [61,62]. For TPVR, these findings support defining a specific support–degradation–remodeling window in which anchoring, sealing, and leaflet support are preserved until tissue incorporation is sufficient, followed by gradual withdrawal of mechanical constraint without recoil, migration, or valve incompetence.

No single degradable metal currently satisfies all TPVR requirements: longitudinal porcine data show that loss of structural integrity, peak inflammatory activity, and near-complete material disappearance can occur at different time points for magnesium devices [63]. Zinc alloys may provide a more moderate degradation profile, but recent long-term porcine and micro-CT studies indicate that ion-release kinetics, degradation uniformity, and local tissue responses depend strongly on alloy composition and the implantation environment [64,65].

Future TPVR devices are therefore likely to require alloy optimization, multilayer or spatially selective coatings, graded strut geometry, or hybrid structures rather than a homogeneous monolithic frame. These strategies should control where and when support is lost, instead of merely reducing the mean corrosion rate.

**Figure 3:**
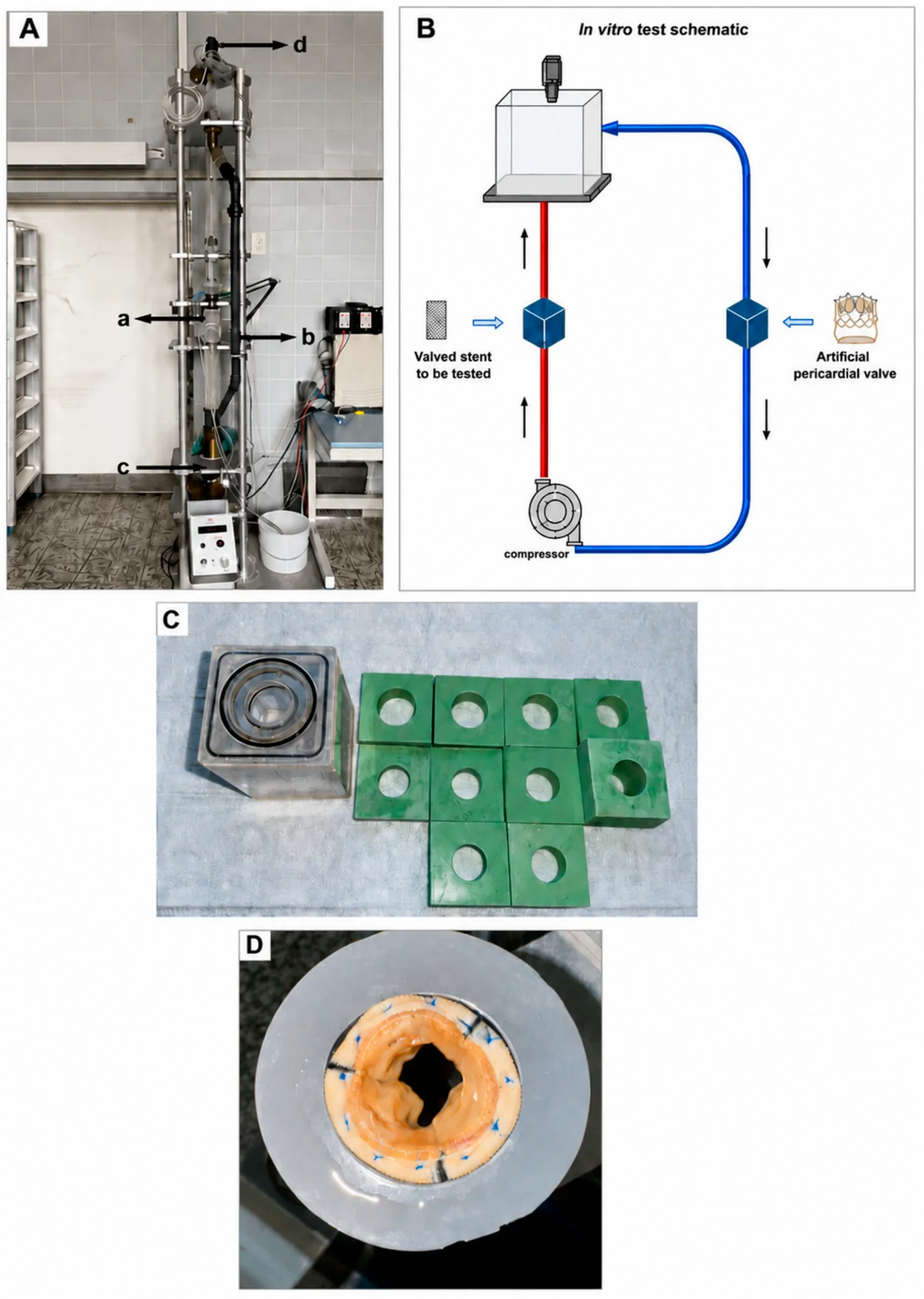
In vitro hydrodynamic testing system. (**A**) Overall view of test devices, a: position for the valved stent to be tested; b: opposing pericardial valve position; c: compressor used to simulate the cardiac cycle; d: the camera view; (**B**) The operating principle of the test system; (**C**) Cubical container for securing the valved stent; (**D**) Opposing pericardial valve prosthesis.

The marked anatomical variability of the RVOT also argues for anatomy-specific frame design. Patient-derived imaging, finite-element analysis, and fluid–structure interaction modeling can be used to optimize anchoring zones, local strut density, expansion symmetry, and leaflet coaptation [40,59]. The key advance will be to incorporate time-dependent material behavior: deployment injury, cyclic fatigue, corrosion, tissue ingrowth, and declining radial support should be modeled as interacting processes rather than independent endpoints. Bench studies, including pulse-duplicator systems such as that shown in Figure 3, remain necessary, but future protocols should assess retained radial force, fatigue resistance, and hydrodynamic performance at several predefined stages of degradation rather than only in the intact device [66,67,68,69,70].

Frame degradation must also be coordinated with leaflet adaptation and tissue remodeling. Recent regenerative-valve studies demonstrate host-cell recruitment, extracellular-matrix formation, and development of organized valve-root tissue but also identify incomplete leaflet cellularization, persistent foreign-body responses, leaflet thickening or retraction, and focal structural defects as potential failure modes [71,72]. Prototype polycaprolactone (PCL)-based tri-leaflet valved stents further demonstrate the feasibility of integrating biodegradable leaflet matrices with transcatheter constructs [73]. A disappearing frame will provide a clinical advantage only if the leaflet–frame interface has achieved sufficient biological and mechanical stability. Future designs should therefore combine controlled frame degradation with strategies that promote endothelialization, guide macrophage-mediated remodeling, limit thrombosis and calcification, and preserve leaflet geometry.

Dedicated large-animal studies should extend beyond the expected period of complete frame resorption and include both constrained landing zones and native RVOT models. Evaluation should include migration, paravalvular leakage, fatigue fracture, residual degradation products, local and systemic inflammation, leaflet remodeling, endocarditis, and the feasibility of subsequent reintervention, in addition to transvalvular gradient and regurgitation. Growing-animal models will be particularly important for determining whether resorption permits meaningful accommodation of somatic growth. A cautious first-in-human pathway may initially target patients with predictable landing zones, such as dysfunctional right ventricle-to-pulmonary artery conduits or surgical bioprostheses, before expansion to large and irregular native RVOT anatomies [2,3].

Overall, progress will depend on integrating frame composition, local geometry, degradation kinetics, leaflet biology, and patient anatomy into a temporally coordinated valve system; the decisive benchmark will be sustained valve function before, during, and after resorption, rather than complete disappearance alone.

## 9. Conclusions

Coronary BRS studies have shown that successful resorbable stents require more than biodegradation; they must provide adequate support, controlled degradation, and favorable tissue healing. TPVR is relevant for this concept because many patients are young and require repeated interventions. Mg-Zn alloys offer a promising and adjustable material platform.

## Figures and Tables

**Table 1:** Clinical applications of polymeric and metallic bioresorbable coronary stents.

Trial	Stent/Material	Strut Thickness	Procedure/Regimen	Mean Age	Follow-Up	Main Findings	Ref.
ABSORB III	Absorb BVS/PLLA everolimus-eluting scaffold	150–157 μm	PCI; DAPT for at least 12 months	63.5 ± 10.6 years	3 years	Higher TLF than metallic EES (13.4% vs. 10.4%) and higher definite/probable ScT (2.3% vs. 0.7%).	[16]
ABSORB randomized trials pooled analysis	Absorb BVS/PLLA everolimus-eluting scaffold	150–157 μm	PCI; trial-specific DAPT	63 years	3 years	Higher TLF than metallic EES (11.7% vs. 8.1%) and higher definite/probable ScT (2.4% vs. 0.6%).	[6]
FANTOM II	FANTOM/iodinated tyrosine polycarbonate sirolimus-eluting scaffold	125 μm	PCI; DAPT per protocol	62.7 ± 10.1 years	1 year	Improved radiopacity and thinner struts; low early adverse event rate; definite/probable ScT about 0.4%.	[19]
BIOSOLVE-II	Magmaris/DREAMS 2G Mg alloy sirolimus-eluting scaffold	150 μm	PCI; DAPT generally recommended for 6 months	65.2 ± 10.3 years	5 years	Sustained long-term safety; TLF 8.0%; no definite/probable ScT reported.	[24]
BIOSOLVE-IV	Magmaris/Mg alloy sirolimus-eluting scaffold	150 μm	PCI; DAPT per protocol	62.0 ± 11.0 years	12 months	Real-world registry; TLF 4.3%; low definite/probable ScT, approximately 0.5%.	[25]
IBS-FIM	IBS coronary scaffold/nitrided iron sirolimus-eluting stent	53 μm	PCI; DAPT per protocol	53.2 years	3 years	Ultrathin iron stent; TLF 6.7%; no definite/probable ScT; slow degradation remains a key limitation.	[26]

Abbreviations: BVS, bioresorbable vascular scaffold; DAPT, dual antiplatelet therapy; EES, everolimus-eluting stent; IBS, iron bioresorbable scaffold; PCI, percutaneous coronary intervention; PLLA, poly-L-lactic acid; ScT, scaffold thrombosis; TLF, target lesion failure.

**Table 2:** Preclinical vascular studies of bioresorbable metallic stents in animal models.

Stent/Material	Animal Model	Year	Follow-Up	Main Findings	Ref.
Early magnesium alloy stent	Porcine coronary artery	2003	56 days	Demonstrated the feasibility of magnesium-based absorbable stenting, but rapid degradation and limited mechanical persistence were major concerns.	[29]
Zn-Cu biodegradable stent	Porcine coronary artery	2019	6, 12, 18, and 24 months	Maintained early structural integrity; progressive degradation; approximately 28% residual stent volume at 24 months; no severe inflammatory response.	[27]
PDLLA/rapamycin-coated JDBM scaffold (Mg-Nd-Zn-Zr)	Porcine coronary artery	2021	6 months	Demonstrated acceptable biosafety and antirestenotic efficacy; inhibited neointimal hyperplasia and promoted re-endothelialization.	[28]

Abbreviations: JDBM, Mg-Nd-Zn-Zr magnesium alloy system; PDLLA, poly-D, L-lactic acid.

**Table 3:** Differences between coronary BRS and TPVR BRS.

Parameter	Coronary BRS	TPVR BRS
Target size	2.5–4.0 mm	20–30 mm or larger
Main function	Maintain vessel patency	Valve anchoring and leaflet support
Key risk	Thrombosis, restenosis	Migration, fatigue fracture, valve dysfunction
Degradation goal	Vessel healing	Valve integration and tissue remodeling
Major material requirement	Thin struts, anti-thrombosis	Ductility, radial force, fatigue resistance

Abbreviation: bioresorbable stents (BRS).

**Table 4:** TPVR-oriented comparison of bioresorbable stent materials.

Material Class	Representative Examples	Potential Advantage for TPVR	Main Limitation
Polymeric stents	PLLA, tyrosine polycarbonate, PCL-based matrices	Useful for leaflets, coatings, drug delivery, and tissue-engineering matrices	Limited radial strength and ductility as a load-bearing frame
Iron-based alloys	Pure Fe, nitrided Fe, Fe-Mn systems	High strength and thin-strut feasibility	Very slow degradation and possible long-term retention of corrosion products
Magnesium-based alloys	WE43, Mg-rare earth alloys	Good biocompatibility and established clinical experience in coronary BRS	Rapid corrosion and possible early loss of radial support
Zinc-based alloys	Zn-Cu, Zn-Mn, Zn-Ag	Intermediate corrosion rate and favorable preclinical vascular response	Pure Zn is weak; alloy fatigue data remain limited
Mg-Zn-containing alloys	Mg-Nd-Zn-Zr, Mg-Zn-Y-Nd, Mg-Zn-Ca systems	Tunable strength, ductility, and corrosion behavior; favorable vascular healing signals	Requires optimized alloying, coating, and degradation-fatigue testing

**Table 5:** Representative evidence relevant to bioresorbable and regenerative pulmonary valve platforms.

Study	Platform	Route	Model/Cohort	Main Contribution
Shinoka et al. [53]	Biodegradable scaffold-based valve leaflet	Surgical	Lamb	Early proof of scaffold-guided pulmonary valve tissue formation
Cebotari et al. [55]	Decellularized pulmonary allograft	Surgical	Children and young adults	Early clinical evidence for remodeling-oriented pulmonary valve replacement
Sarikouch et al. [57]	Decellularized fresh pulmonary homograft	Surgical	Clinical cohort	Decade-long clinical experience with decellularized pulmonary homografts
Driessen-Mol et al. [56]	Off-the-shelf tissue-engineered heart valve	Transcatheter	Sheep	Demonstrated catheter-based implantation and remodeling in the pulmonary position
Kluin et al. [58]	Bioresorbable elastomeric valve implant	Preclinical implant	Sheep	Demonstrated 12-month in situ remodeling and extracellular matrix formation
Emmert et al. [59]	Computationally optimized TEHV	Preclinical	Sheep	Linked scaffold design with long-term in vivo performance

Abbreviations: TEHV, tissue-engineered heart valve.

## Data Availability

No new data were created or analyzed in this study. Data sharing is not applicable to this article.
